# Mass flux decay timescales of volcanic particles due to aeolian processes in the Argentinian Patagonia steppe

**DOI:** 10.1038/s41598-020-71022-w

**Published:** 2020-09-02

**Authors:** Lucia Dominguez, Eduardo Rossi, Leonardo Mingari, Costanza Bonadonna, Pablo Forte, Juan Esteban Panebianco, Donaldo Bran

**Affiliations:** 1 Department of Earth Sciences, University of Geneva, Geneva, Switzerland; 2 Barcelona Supercomputing Center, Barcelona, Spain; 3 Instituto de Estudios Andinos (IDEAN) (UBA–CONICET), Buenos Aires, Argentina; 4 INCITAP (Institute for Earth and Environmental Sciences, CONICET) and Facultad de Ciencias Exactas y Naturales, UNLPam, Santa Rosa, Argentina; 5 National Institute of Agricultural Technology (INTA), Bariloche, Argentina

**Keywords:** Volcanology, Atmospheric dynamics

## Abstract

We investigate the timescales of the horizontal mass flux decay of wind remobilised volcanic particles in Argentina, associated with the tephra-fallout deposit produced by the 2011–2012 Cordón Caulle (Chile) eruption. Particle removal processes are controlled by complex interactions of meteorological conditions, surface properties and particle depletion with time. We find that ash remobilisation follows a two-phase exponential decay with specific timescales for the initial input of fresh ash (1–74 days) and the following soil stabilisation processes (3–52 months). The characteristic timescales as a function of particle size shows two minimum values, identified for sizes around 2 and 19–37 $\upmu$m, suggesting that these size-range particles are remobilised more easily, due to the interaction between saltation and suspension-induced processes. We find that in volcanic regions, characterised by a sudden release and a subsequent depletion of particles, the availability of wind-erodible particles plays a major role due to compaction and removal of fine particles. We propose, therefore, a simple and reproducible empirical model to describe the mass flux decay of remobilised ash in a supply-limited environment. This methodology represents an innovative approach to link field measurements of multi-sized and supply-limited deposits with saltation erosion theory.

## Introduction

**Figure 1:**
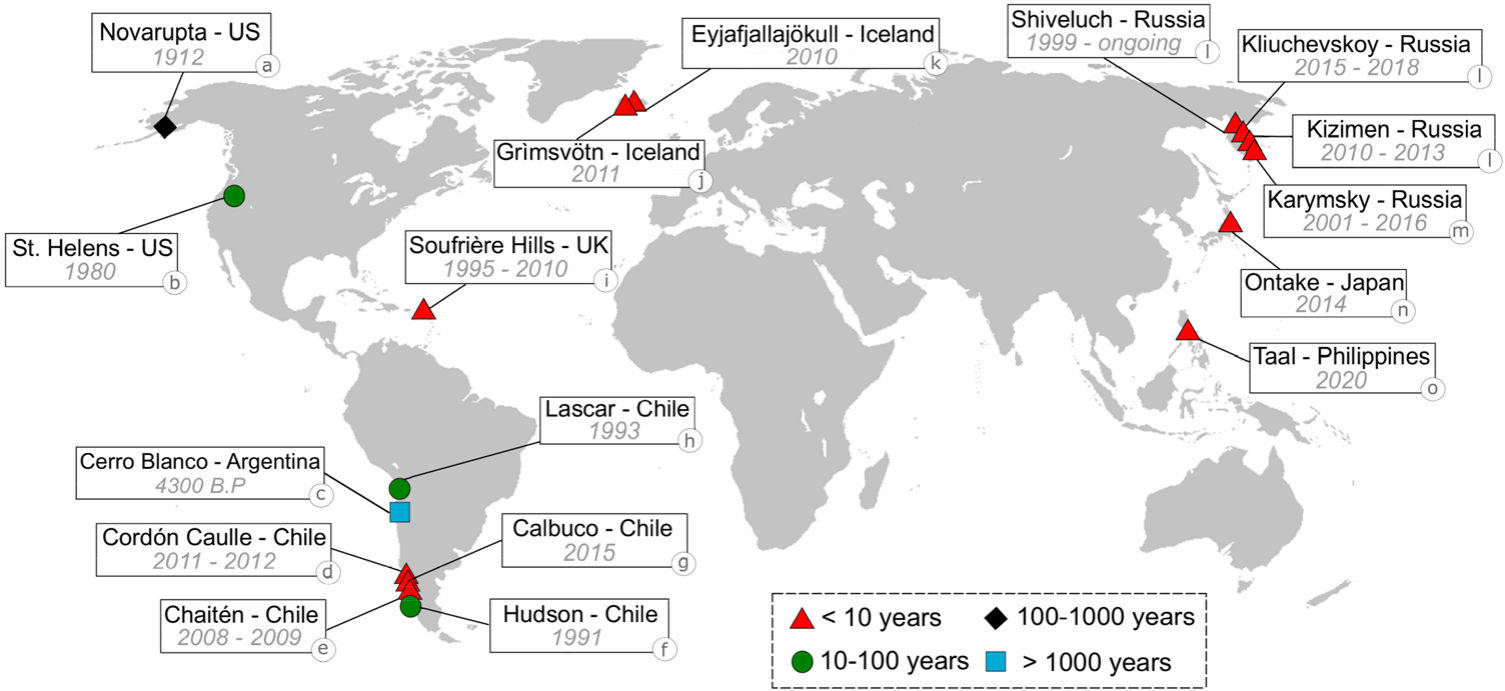
Global distribution of major wind-remobilisation events associated with pyroclastic deposits reported in literature. Source volcanoes and eruption years are indicated. Colours show time elapsed between the occurrence of the eruption and the last remobilisation event reported. Major events are associated with several kilometres of extension, usually detected by satellite imagery. Even though they can represent a major concern for exposed communities, small events are not reported here as they are either not recorded or recorded in local or national media and are understudied. References: (**a**) Novarupta^1,2,11,12^, (**b**) Mount Saint Helens^13^, (**c**) Cerro Blanco^3,4^, (**d**) Cordón Caulle^14–23^, (**e**) Chaitén^19^, (**f**) Hudson^14,24,25^, (**g**) Calbuco^26^, (**h**) Lascar^4^, (**i**) Soufrière Hills^27^, (**j**) Grímsvötn^28^, (**k**) Eyjafjallajökull^8,28–31^, (**l**) Shiveluch, Kliuchevskoy and Kizimen^32^, (**m**) Karymsky^32,33^, (**n**) Ontake^34^, (**o**) Taal^35^. Map created by the authors using ArcGIS software by Esri (version 10.3 copyright 1995–2015) used herein under license. World map background datasource: https://www.arcgis.com/home/item.html?id=ad61dcd7dd244d4096c22a49cc97011f.

Wind-remobilisation phenomena in volcanic regions result from a complex combination of meteorological conditions, surface properties and the intrinsic features of pyroclastic deposits at different temporal and spatial scales. Due to the continuous variation of wind intensity and direction, transport and deposition of remobilised ash are intermittent but long-lasting processes, affecting areas larger than those initially impacted by the primary emplacement of volcanic deposits (mostly deposits associated with tephra fallout and pyroclastic density currents). These processes can last from days to millennia after the eruption, generating significant impacts on communities over extensive areas (particularly associated with human and animal health, reduced visibility for air and road traffic, and ash contamination of crops) (Fig. 1). A typical example is represented by the continuous remobilisation events of the 1912-Novarupta deposit (Katmai, USA), whose clouds can rise up to 3.5 km above sea level with an extension of up to 250 km far from the original source^1,2^. Another striking example is the remobilisation event reported by the Volcanic Ash Advisory Centre (VAAC) of Buenos Aires in 2015 associated with the 4.5 ka Cerro Blanco deposit accumulated in the Fiambalá Basin (Argentina), whose ash clouds of about 5-6 km height dispersed towards the East, up to 800 km far from the basin^3,4^. Interestingly, United Nations global studies have catalogued Iceland and Patagonia as locations of moderate to very high potential areas of dust emission, according to a dataset of sand and dust storms from 1974 to 2012^5,6^. These studies consider anthropogenic activities and presence of playa lakes rather than volcanic sources, and do not include the latest events related to the 2011–2012 Cordón Caulle and 2015-Calbuco (Chile) eruptions. However, more detailed studies have demonstrated the significant contribution of volcanic sources to the particulate matter in Iceland^7–9^ and Patagonia^10^.

The contrasting timescales over which ash-remobilisation occurs (i.e., syn-eruptive to millennia after the eruption) are controlled by factors usually difficult to determine, such as the availability of loose material (i.e., an almost instantaneous injection of a large amount of particles) and the change in compaction of the primary deposit over time (i.e., relatively rapid compaction due to water and/or human actions), which make erosion of volcanic deposits different to that of mineral sand and dust. Aeolian remobilisation of volcanic ash is typically studied within the framework of dust emission schemes and the saltation theory of particles^17,21,36,37^. In this study we explore temporal variation of the streamwise (horizontal) mass flux *Q* of volcanic particles of the tephra-fallout deposit in Argentina produced by the 2011–2012 eruption of Cordón Caulle volcano. The streamwise mass flux *Q* is defined as the vertical integral of the streamwise mass flux density *q* measured at each height *z*^38–42^. In this paper with Q we indicate *streamwise mass flux* and with q we indicate *mass flux* (see "Materials and methods" section). Q is predominantly controlled by two competing forces: aerodynamic lift forces and resistive cohesive forces between particles. Mass transport occurs when the wind friction velocity *u*$_{*}$ exceeds the threshold friction velocity *u*$_{*t}$, which depends on both particle Reynolds number and inter-particle cohesive forces. These quantities are difficult to estimate and they are often determined from wind-tunnel experiments^43^. Based on the models of Owen^39^ and Iversen et al.^43^, Shao and Lu^44^ have derived a simple expression for the threshold friction velocity as a function of the size and density of spherical particles, the particle Reynolds number and the cohesive forces. Further corrections to u$_{*t}$ accounting for soil moisture can also be considered through the parameterisation scheme of Fecan et al.^45^. *Q* can then be estimated as a function of *u*$_*$ and *u*$_{*t}$ for mono-sized, dry or wet, loose soils. In fact, these are saltation models, based on experimental data of mono-sized sand transport, which have not been tested for dust particles or mixed grain sizes. However, in natural environments, soils have dispersed sizes and the effects of packing arrangement of particles and compaction of deposits over time, have not yet been well constrained. Since particle interactions remain challenging to model, we follow Shao et al.^46^ and assume that *Q* is not significantly altered by the presence of multi-sized particles. Thus, the total streamwise mass flux can be calculated as a weighted integral of all size fractions in the primary tephra-fallout deposit (i.e., parent soil). (See "Materials and methods" section and ESM-Table S1).

A key difference between aeolian erosion of volcanic deposits and that of sand and dust erosion is the depletion of available material. The previously mentioned theoretical framework assumes an unlimited and constant supply of particles^41,42^. However, in a volcanic context, the supply of particles over time is certainly not constant but depends on eruption magnitude, intensity, duration and frequency. This variable sediment supply leads to complex remobilisation dynamics and deposits at volcanoes with pulsatory and long-lasting activity (e.g., Soufrière Hills volcano, Montserrat, UK; Eyjafjallajökull, Iceland; Fig. 1). Furthermore, following the cessation of primary sedimentation, the erodibility of the deposit decreases with time due to the removal of fine particles and compaction. With the exception of a few studies^47,48^, supply-limited systems have been poorly explored. A better understanding of the mass transport temporal decay is, therefore, required for remobilisation studies in volcanic regions.

**Figure 2:**
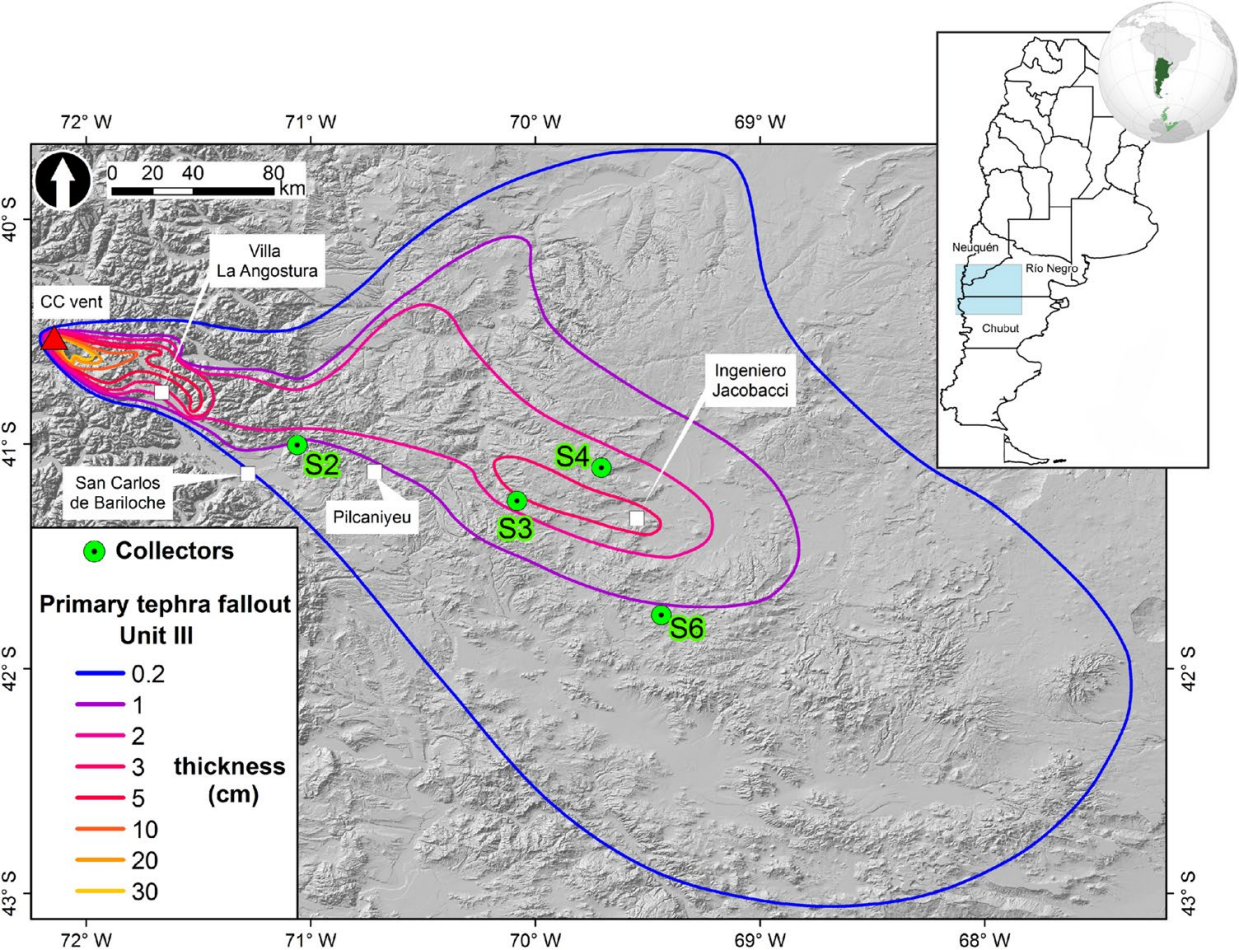
Location of area of study. Green points correspond to collectors of airborne ash considered in this study. Isopach map corresponds to the Unit III of the primary tephra-fallout deposit associated with the 2011–2012 Cordón Caulle eruption (adapted from Dominguez et al.^23^). Map created by the authors using ArcGIS software by Esri (version 10.3 copyright 1995–2015) used herein under license. Hillshade generated from the Global Multi-resolution Terrain Elevation Data (GMTED2010) available in https://earthexplorer.usgs.gov. Globe image available in https://www.pngegg.com/en/png-yzcom.

Here we present a detailed study of the decay of the temporal mass flux of the remobilised airborne material in the Patagonia steppe (Argentina), associated with the primary tephra-fallout deposit produced by the 2011–2012 Cordón Caulle (CC) eruption, through a systematic sampling of remobilised material from 2011 to 2016. Due to the prevailing winds, a vast area in the Argentinian Andes and the Patagonian steppe was covered by $\thicksim$1 $\hbox {km}^3$ of rhyolitic tephra^49–52^. The primary tephra-fallout deposit consists of 4 units of which the first three were deposited during the 6–15 June, 2011 and the Unit IV was deposited only in very proximal areas in Chile after 15 June, 2011. Unit III, being the most widely dispersed, upper and fine-grained, is the unit that was first remobilised^23^ (Fig. 2). However, after Unit III was completely eroded, Unit I and II also started being remobilised, particularly in unprotected areas (e.g., low vegetation cover). We analyse (i) the temporal distribution of the remobilisation processes associated with the Unit III of the CC tephra-fallout deposit, (ii) the mass flux decay timescale as a function of particle size, (iii) the role of meteorological and soil conditions based on the comparison of the measured (*Q*$_{msd}$) and theoretical (*Q*$_{th}$) streamwise mass fluxes, and (iv) the influence of particle supply in volcanic erosion systems. A complementary analysis of the spatial distribution of remobilisation is available in the Electronic Supplementary Material (ESM-Section 1). We show that meteorological fluctuations, material supply, compaction of primary deposit and particle size are key parameters affecting the temporal evolution of the mass flux. Consequently, the current theoretical saltation schemes, which are based on assumptions of unlimited supply and uniform particle size, should be carefully applied to fresh volcanic deposits.

## Results

### Wind-remobilisation of the Cordón Caulle tephra-fallout deposit: temporal distribution

**Figure 3:**
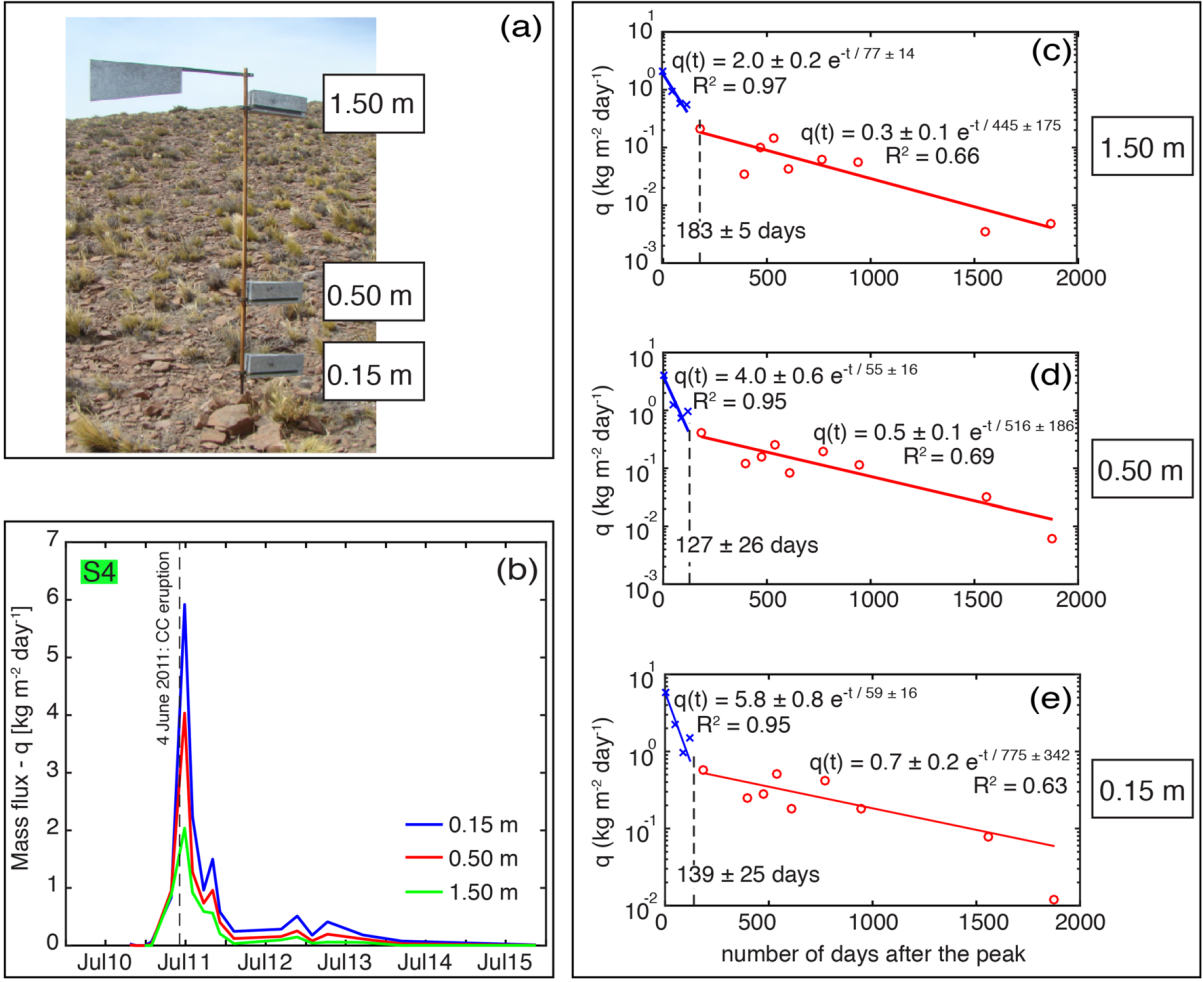
(**a**) Collector with a wind-oriented weather vane constituted by three samplers at fixed heights (more details in the "Materials and methods" section). Photograph taken by Donaldo Bran. (**b**) Mass flux measured at collector S4 for the 3 heights. Dashed line corresponds to the CC-eruption starting date. (**c–e**) Mass flux decays with time for each height at collector S4. Blue (Phase I) and red (Phase II) lines indicate the fitting of a two-phase exponential function. Fitted equations and $\hbox {R}^2$ are shown. Dashed lines indicate the transition between the two stages.

In order to study soil erosion processes, a systematic network of 4 horizontal collectors setup by INTA (National Agricultural Technology Institute of Argentina) in strategic erosion sites of the Patagonia steppe, was operated from 2011 until 2016 (Fig. 2). Each collector consists of 3 dedicated samplers at fixed heights above the ground (Fig. 3a). We calculated mass fluxes from the mass of collected material, the duration of accumulation and the cross-sectional area of the collector (See "Materials and methods" section). Temporal evolution of the mass flux is the result of a complex interaction between the surface and meteorological conditions, and is intrinsically associated with the eruptive evolution and the primary tephra sedimentation. Whilst the climactic eruptive phase started on 4 June 2011 and lasted approximately 24–30 hours, the Unit III was produced and sedimented some days after, between the 6 and 15 of June^52^ (Fig. 2). There are evidences of syn- and post-eruptive remobilisation immediately after the eruption^23^; however, it is impossible to reconstruct all the remobilisation events. Since the largest amount of mass was collected on the site S4 and it represents the most complete sub-dataset, we focus on this single site to illustrate the main features of the temporal mass flux variation. Figure 3b shows that the mass flux decays non-monotonically over time for collector S4. The first peak at this site is recorded on 2 August 2011, two months after the beginning of the eruption. Additionally, secondary peaks were recorded on 6th December 2011, 29th January 2013 and on the 18th September 2013. Posteriorly, mass flux monotonically decreases after April 2014 (Fig. 3b). A complementary analysis of the spatial and temporal evolution at the other collection sites (S2, S3 and S6 of Fig. 2) in relation with the primary tephra-fallout deposit is available in the ESM-Section 1.

### Horizontal mass flux decay of volcanic particles

#### Total mass flux decay

Aeolian processes are strongly influenced by the supply of erodible particles, which has been shown to decrease exponentially with time^41,46,47,53^. Consequently, mass flux *q* as a function of time *t* for each height, can be described as,

1 $$\begin{aligned} q(t) = a e^{\frac{-t}{\tau } }, \end{aligned}$$

where $\tau$ is the decay timescale and *a* is a constant.

A mass flux peak significantly above the background erosion was recorded after the emplacement of the tephra-fallout deposit (streamwise mass flux before the eruption for the same location was estimated as 0.09 kg m^-1^ day ^−1^^21^). Following this peak, we find that the evolution of the mass flux can be described by two successive phases of exponential decay with distinct timescales (Fig. 3c–e). This is true for each collector height (0.15 m, 0.50 m, 1.50 m), with inflection points between the two decay stages at 139 ± 25, 127 ± 26 and 183 ± 5 days, respectively. Decay timescale, $\tau$ (Eq. 1), is also shown for each exponential curve. The two-phase processes represent, therefore, two different timescales with the first, faster phase having shorter timescales (59 ± 16, 55 ± 16, 77 ± 16 days) than the second, slower phase (775 ± 342, 516 ± 186, 445 ± 175 days) for the 3 heights, 0.15 m, 0.50 m and 1.50 m, respectively. These results also show that the slowest decay is observed for the collector at 1.50 m for the first phase, and at 0.15 m for the second phase (Fig. 3c–e).

#### Mass flux decay as a function of particle size

Considering that tephra-fallout deposits have disperse grainsize distributions, we quantify the evolution of mass flux of different size fractions and investigate if different fractions exhibit the same two-phase mass flux decay as the total remobilised material. To this end, the mass collected at site S4 has been analysed for grainsize using half-phi-size classes. This sub-dataset corresponds to 14 collection periods at 3 different heights (see "Materials and methods" section and ESM-Table S3). We fit the mass flux of each size class at each height to a double exponential decay of the form,

2 $$\begin{aligned} q(t) = b e^{\frac{-t}{\tau _1} }+ c e^{\frac{-t}{\tau _2}} \end{aligned}$$

where $\tau _1$ and $\tau _2$ are the fast and slow decay timescales corresponding to phase I and phase II, respectively; *b* and *c* are constants giving the peak mass flux at *t=0*.

**Figure 4:**
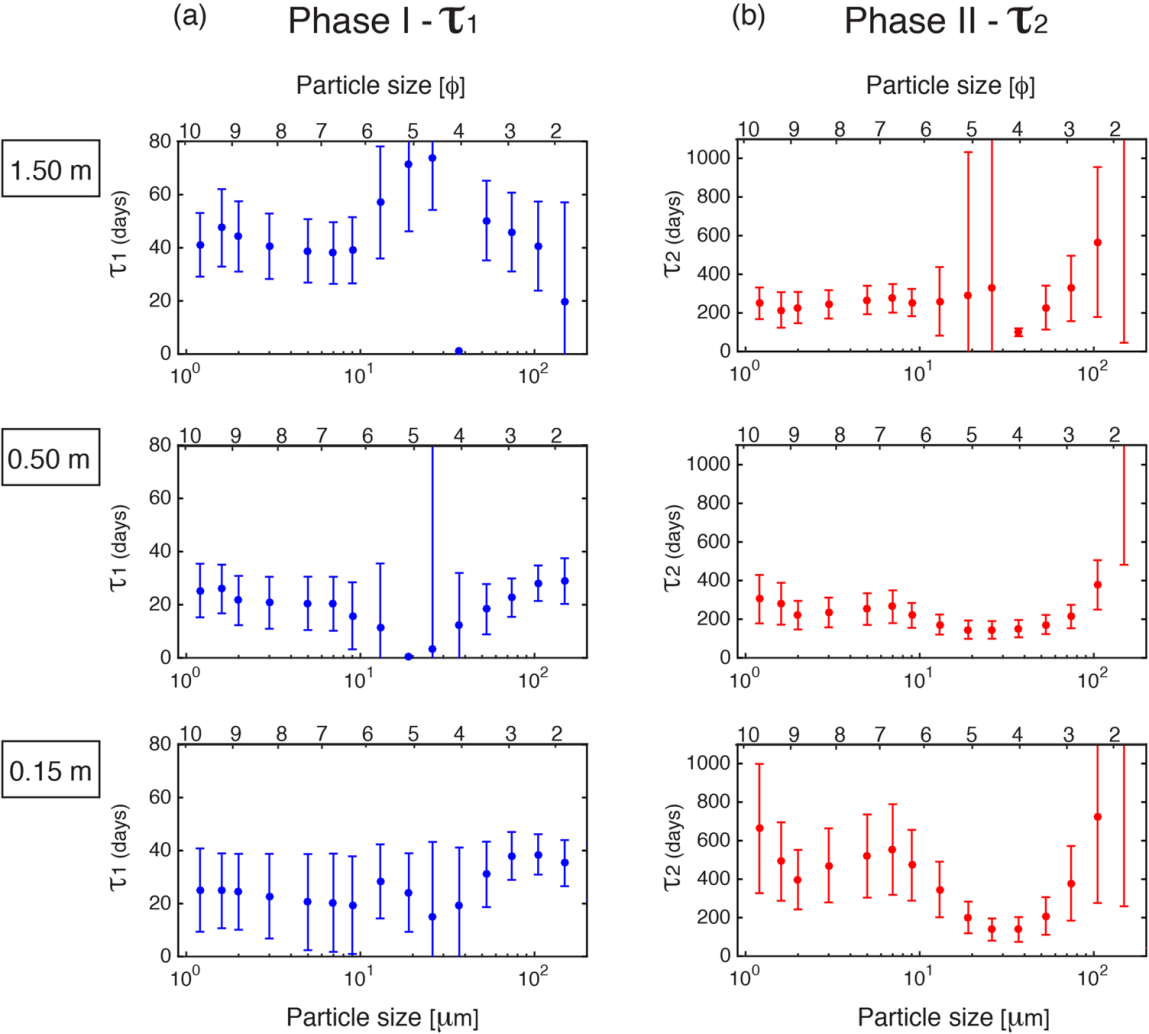
Decay timescales (**a**) $\tau _1$ and (**b**) $\tau _2$ as a function of particle size for the 3 sampling heights (0.15 m, 0.50 m, 1.50 m). Error bars indicate the 68$\%$ confidence interval; uncertainties associated with the first phase are notably larger than those of the second phase. Phi and metric scales are shown; the equivalence between both scales, as well as the representative size values (used hereafter) for each bin are presented in the ESM-Table S2. Fitting procedure details are presented in the "Materials and methods" section.

Mass flux decay timescales, expressed by $\tau _1$ and $\tau _2$, provide an insightful description of erosion phenomena when displayed as a function of particle size (Fig. 4). Phase I decay timescales are all within the range of $\backsim$1–74 days (Fig. 4a), whereas phase II decay timescales are typically one-two orders of magnitude slower ($\backsim$3-52 months, Fig. 4b). Similarly to the whole mass (Fig. 3b), the slowest decay for the phase I and the fastest decay for the phase II are associated with the 1.50 m height for all the size fractions. Whilst mass flux decay timescales show a clear trend for the phase II at all the heights, this trend is less notable for phase I, for the 0.15 and 1.50 m measurement heights. Consequently, $\tau _2$ shows a robust and distinct U-shaped curve at all heights with two minima values: at 2 $\upmu$m and more prominently at 19-37 $\upmu$m (Fig. 4b). In contrast, $\tau _1$ exhibits a weak minimum from 19 to 37 $\upmu$m slightly varying with height (Fig. 4a). Following sections present how the meteorological, surface parameters and material supply can influence this temporal variation.

### Role of meteorological conditions and material supply on the streamwise mass flux

**Figure 5:**
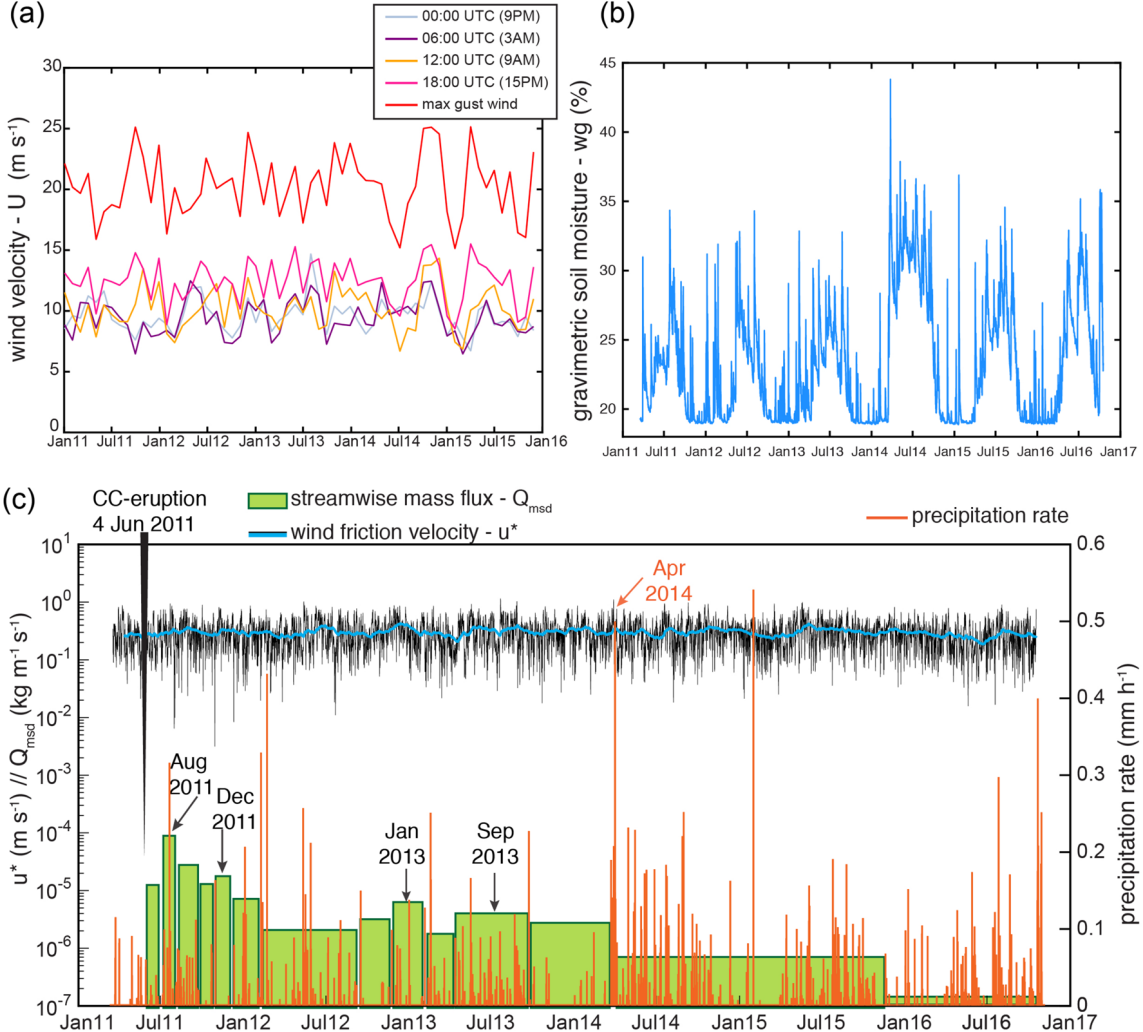
Role of meteorological conditions on the streamwise mass flux (**a**) Monthly average values of wind velocity at different day times, local time is shown in parenthesis. (**b**) Gravimetric soil moisture. (**c**) Measured streamwise mass flux *Q*$_{msd}$ for the total mass at S4, wind friction velocity *u*$_*$ and precipitation rate *p*, over time. Dates in black indicate the time when peak *Q*$_{msd}$ values are recorded (Fig. 3b). Meteorological and surface data from the ECMWF ERA-Interim Re-Analysis dataset (European Center for Medium-range Weather Forecasts).

The frequency and intensity of ash-remobilisation events are related to specific meteorological variables (e.g., surface wind velocity, precipitation, low-level and atmospheric humidity) and surface features (e.g., vegetation cover, soil moisture, grainsize and density of particles)^38,39,41,42^. In the Argentinian Patagonia, recurrent wind remobilisation is associated with the months of austral spring and austral summer^17,22^, due to the strong winds and gusts that can reach up to  90 km $\hbox {hr}^{-1}$ (Fig. 5a). Local observations (informal interviews in Ingeniero Jacobacci, Fig. 2) suggest that events are more frequent during the afternoon, particularly after midday, which coincides with the strongest winds (> 36 km $\hbox {h}^{-1}$, Fig. 5a) and high surface temperatures on the ground. Maximum gust values are mainly recorded during the summer ($\sim$90 km $\hbox {h}^{-1}$, Fig. 5a). The prevailing surface wind direction at surface is W to E^23^. A strong seasonality in soil moisture is evidenced by lower values of gravimetric soil moisture during spring and summer (19–26$\%$) than in autumn and winter (21–35$\%$) periods (Fig. 5b). There was a notable increase in soil moisture since the winter in 2013, peaking in spring 2014 (Fig. 5b).

In addition to these meteorological conditions, the measured streamwise mass flux *Q*$_{msd}$ calculated as the vertical integral of the mass flux *q* is shown in the Fig. 5c. The four mass flux peaks in August 2011, December 2011, January 2013 and September 2013, that were recorded by the individual collectors (Fig. 3b) are also present in the integrated signal. In order to better understand the influence of each meteorological and soil parameter, and the importance of the availability of particles on the mass flux decays, we analyse the temporal evolution by comparing the measured *Q*$_{msd}$ and the theoretical *Q*$_{th}$ streamwise mass fluxes.

#### Comparison of measured and theoretical streamwise mass fluxes: impact of a finite supply of particles

In order to calculate *Q*$_{th}$, we first use the model of Shao and Lu^44^ to estimate the threshold friction velocity *u*$_{*t}$, considering a $\gamma$ of 1.65 $\times$  10$^{-4}$ kg $\hbox {s}^{-2}$, which fits best the airborne material analysed in this study (ESM-Fig. S2). Secondly, we account for soil moisture through the parameterisation of Fecan et al.^45^. We combine then the resulting *u*$_{*t}$ with the wind friction velocity, *u*$_*$, to calculate *Q*$_{th}$ using the saltation model of Owen^39^ for all particle sizes. Two meteorological conditions have been considered in order to constrain the theoretical streamwise mass flux: (i) the precipitation rate must not be higher than 0.01 mm $h^{-1}$, according to the UK Met Office strategy^30^; and (ii) *u*$_*$ must exceed *u*$_{*t}$ for each particle size (See "Materials and methods" section).

This theoretical framework assumes that saltation occurs under an infinite supply of particles^41,47^. However, in volcanic regions characterised by an almost instantaneous release of a large amount of particles and its subsequently depletion, the supply of material is limited. This depletion creates the condition known as supply-limited saltation or source-limited saltation^41^. We account then for a correction factor on the supply-limited streamwise mass flux *Q*$_{slm}$ based on the temporal evolution of the erodibility, $\sigma$, or the potential of the surface to be eroded^41^, according to,

3 $$\begin{aligned} Q_{slm} = \sigma (t) \, Q_{th} \end{aligned}$$

**Figure 6:**
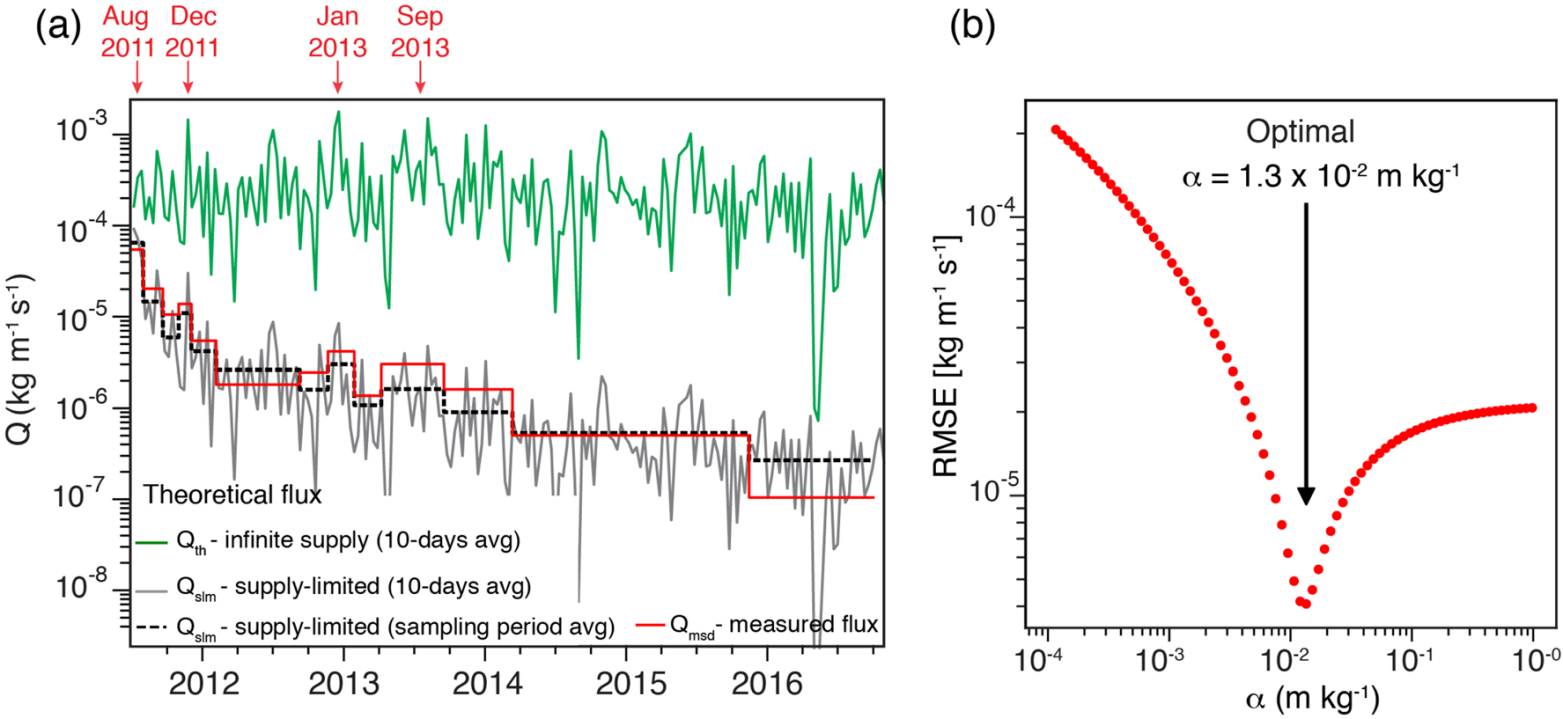
Model for the streamwise mass flux assuming a time-dependent erodibility to account for a supply-limited source of particles. (**a**) Comparison of measured *Q*$_{msd}$ (in red) and theoretical streamwise mass fluxes *Q*$_{th}$ for infinite-supply (in green) and supply-limited *Q*$_{slm}$ (in grey and dotted black) cases. Dates in black indicate the time when peak *Q* values are recorded (Fig. 3b). (**b**) Root mean square error between *Q*$_{msd}$ and *Q*$_{slm}$ for different values of $\alpha$, the optimal being at 1.3 $\times$  10$^{-2}$ m $\hbox {kg}^{-1}$.

We first assume a constant erodibility value of $\sigma$ = 0.21 following the numerical simulations of Mingari^54^ for the outstanding event of ash resuspension in October 2011 associated with the 2011–2012 CC eruption. Figure 6a shows the comparison of the measured *Q*$_{msd}$ for the 14 collection periods, and the theoretical *Q*$_{th}$ for a 10-day average period, considering an infinite-supply of particles. A substantial disagreement between *Q*$_{msd}$ and *Q*$_{th}$ is evident. Whilst *Q*$_{th}$ remains relatively constant with slight variations due to the meteorological fluctuations (i.e., wind speed and precipitation), *Q*$_{msd}$ decreases with time by more than two orders of magnitude from period 1 to 14. These results demonstrate that the decay of *Q*$_{msd}$ might be intrinsically related to a decreasing supply of particles, and, therefore, $\sigma$ cannot be constant through time. To account for this depletion, we propose the following empirical model to estimate the temporal variability of the erodibility, $\sigma (t)$, according to,

4 $$\begin{aligned} \sigma (t)=\sigma _o \exp \left( -\alpha \int _{t_o}^t Q_{slm}\,dt\right) \end{aligned}$$

where $\sigma _0$ corresponds to the initial erodibility (here, 0.21) and $\alpha$ is an empirical constant parameter with units of (m $\hbox {kg}^{-1}$). The Eqs. (3) and (4) were solved using an iterative approach in order to reconstruct the time series for the supply-limited streamwise mass flux *Q*$_{slm}$. In order to determine the optimal value of $\alpha$, the root mean square error between *Q*$_{msd}$ and *Q*$_{slm}$ for different values of $\alpha$ was computed (Fig. 6b). The best agreement between the empirical model and the measured streamwise fluxes was obtained for $\alpha = 1.3 \times 10^{-2}$ m $\hbox {kg}^{-1}$. The resulting *Q*$_{slm}$, considering a time-dependent erodibility and the optimal $\alpha$, for a 10-day average and for the sampling period average are shown in the Fig. 6a. There is a very good agreement between the measured and the modelled mass fluxes, except for the last sampling period where the predicted *Q*$_{slm}$ is overestimating the measured *Q*$_{msd}$. Interestingly, this model can predict the peaks in August 2011, December 2011, January 2013 and September 2013, previously discussed (Figs. 3b, 5c and 6a).

## Discussion

The temporal variation of ash-remobilisation associated with the 2011–2012 CC eruption highlights important aspects concerning aeolian processes of fresh pyroclastic deposits. Analysis of the mass flux peak values (Fig. 3b) shows that seasonal variability had a strong influence on the frequency of ash remobilisation events and, hence, on the recorded mass flux peaks. Major remobilisation (first and maximum peak) occurred some time after the emplacement of the tephra-fallout deposit associated with the main eruptive phases (4–15 June 2011). The eruption started in the middle of austral winter when high values of precipitation, relative humidity and soil moisture, inhibit aeolian erosion. An important turning point towards a monotonic depletion of mass fluxes since 2014 is associated with a strong precipitation event on 2–8 April 2014 (Fig. 5c). Forte et al.^22^ also reported a decrease on the frequency of remobilisation events since 2014 based on in-person interviews. This precipitation event most likely contributed to erode the primary deposit by surface water flow and also to increase the compaction due to increased soil moisture (Fig. 5b). Whilst the tephra-fallout deposit was produced and deposited within a few days during the eruption (4–15 June 2011), remobilisation processes continue up to the time of writing (2020).

The temporal decay of mass flux is unequivocally not linear. A two-phase decay process is described by a double exponential fitting of the measured mass fluxes, with two strongly contrasting decay timescales (Eq. 2, Figs. 3c–e and 4). The significant mass flux peak due to the sudden large injection of volcanic ash in the system would be neglected by using only a single exponential fit. Indeed, this behaviour is of particular importance in volcanic regions since they are characterised by large amounts of material entering “instantaneously” into the erosional system once a deposit is emplaced. As a consequence, there is a sharp distinction between the first phase, clearly associated with the input of fresh volcanic ash, and the second phase, where deposit stabilisation processes may be developing. Despite the large uncertainty associated with the timescales (see parameters in the Fig. 3c–e), we find that the transition point between the two-phases based on measurement of trapped sediment at 0.15 m and 0.50 m above ground level occurs approximately 5 to 7 months after the eruption onset, whilst this transition occurs 8 months after the eruption for the 1.50 m sampling height. These periods coincide with the end of austral spring and the beginning of austral summer (i.e., October–January), characterised by strong winds and low soil moisture, that might have contributed to a rapid depletion of material due to intense remobilisation. Interestingly, a similar period of about 4–6 months of intense remobilisation was reported by Wilson et al.^14^ after the eruption of the Hudson volcano in August 1991. This faster shift of phases at levels below 0.50 m may correspond to the height of the saltation layer where major particle transport occurs^41^. Additionally, we find that, for the total mass at the three sampling heights, the two timescales are orders of magnitude different: a faster $\tau _1$ of 59 ± 16, 55 ± 16, 77 ± 16 days, and a slower $\tau _2$ of 775 ± 342, 516 ± 186, 445 ± 175 days, at 0.15 m, 0.50 m and 1.50 m, respectively. We noticed that, whilst a slower decay is associated with the 1.50 m in the first phase, it is opposite in the second phase (slower at 0.15 m). This trend, as a function of height for the total mass (Fig. 3), is also confirmed by individual particle size classes (Fig. 4). Although we cannot identify a dominant transport mechanism (such as saltation or suspension^38^), these results suggest that a large fraction of the particles in transport occurred at low levels (< 0.50 m) during the first phase ($\backsim$June 2011–January 2012), while a larger fraction of the particles in transport shifted to a higher level (> 0.50 m) during the second phase ($\backsim$February 2012–December 2016).

Mass flux decay timescales are strongly dependent on particle size. Figure 4 shows that the mass fluxes of particles of different sizes decay at different timescales. We expect that decay timescales are related to the particle threshold friction velocity *u*$_{*t}$ since this physical quantity is the minimum friction velocity at which particle motion starts. It has previously been demonstrated that *u*$_{*t}$ as a function of particle size is described by an U-shaped curve with a minimum for particle sizes around 75–100 $\upmu$m^55^, where inter-particle forces are approximately equal to the gravitational force^42^. For particles smaller than approximately 75 $\upmu$m, *u*$_{*t}$ rapidly increases as particle size decreases due to the increasing strength of cohesive forces; in contrast, for particles greater than approximately 100 $\upmu$m, *u*$_{*t}$ increases due to their larger weight^44,55,56^ (ESM-Fig. S2). Interestingly, we find the same U-shaped trend between the timescale of decay in mass flux and particle size suggesting that this methodology may be extremely useful in linking the saltation theory of particles with field measurements in natural environments. The complexity of natural environments, however, requires a careful interpretation. The contribution of volcanic particles dominant during the first phase is mainly controlled by the size classes present in the primary volcanic deposit, whilst the second phase is likely controlled by a more general trend of erosion in this region with a mixture of the CC volcanic particles, old sediments and soil. In particular, particles of 19 to 37 $\upmu$m diameter, corresponding to the most abundant classes in volume in the primary deposit (ESM-Table S4), show a faster mass flux decay in both phases, although this trend is much more clear for the phase II. Indeed, $\tau _2$ as a function of particle size displays a distinct U-shaped trend, very similar to the theoretical *u*$_{*t}$ trend, but shifted towards a minimum for a particle size between 19 and 37 $\upmu$m instead of 75–100 $\upmu$m particles. This discrepancy could be due to the fact that theoretical *u*$_{*t}$ curve is based on experiments of individual particle sizes and, thus, it does not account for the interaction between particles in a multi-sized distribution, as it is the case of volcanic deposits. Additionally, the lack of reliable experimental data for particles smaller than 50 $\upmu$m^41^ means that the theoretical value of *u*$_{*t}$ is poorly constrained for this size range. Given the high complexity, the effect of grainsize wind segregation (resulting in good sorting) and the interaction between saltation and suspension-induced processes (i.e., splashing generating short or long-term suspension of fine particles^41,42^), are not considered in the theoretical *u*$_{*t}$. In other words, the timescales reflected from field measurements are related to a dynamic threshold friction velocity, whilst the theoretical *u*$_{*t}$ is estimated for particles initially at rest (static threshold friction velocity). Indeed, the distinct trend of $\tau _2$ with 2 minima values at 2 $\upmu$m and 19–37 $\upmu$m might be associated with the effects of complex saltation and suspension-induced transport mechanisms in a supply-limited system where the particles with shorter decay timescales (i.e., low $\tau$) can be remobilised more easily until the total depletion.

Therefore, a crucial parameter in aeolian processes of volcanic deposits is the removal rate of particles. The comparison of *Q*$_{th}$ with *Q*$_{msd}$ clearly shows that measured mass fluxes become much lower than the theoretical estimates with time (Fig. 6a). Since the theoretical saltation models assume an infinite supply of particles, the variations in *Q*$_{th}$ are only associated with meteorological fluctuations (e.g., seasonality). Even though parameters that also modulate particle flux have not been considered in this study due to the complexity of their numerical analysis (e.g., vegetation, surface crust development, gusts or snow), their influence likely follow seasonal variations as observed by Shinoda et al.^57^ in temperate grasslands. However, it has been demonstrated that, in this study, *Q*$_{msd}$ decays exponentially with time (Figs. 3, 4, 5c and 6a). These results suggest that, although meteorological conditions play an important role in aeolian processes, the exponential variation of mass transport of volcanic particles with time is substantially governed by the amount of available material in the long-term (i.e., months to years), as it has also been observed in supply-limited studies for loess deposits^47^, numerical simulations^48^, and experiments^53^.

The limitation on material supply might be mainly related to the compaction of the primary volcanic deposit, the assimilation of ash by vegetation, and the loss of fine material with time, all processes being very difficult to numerically quantify. One possibility to account for compaction is through the cohesive forces described by the parameter $\gamma$ of the Shao and Lu model^44^. However, this scheme assumes that cohesive forces are linearly proportional to the particle diameter, but the effect of moisture and chemical bindings are ignored. An alternative approach to consider the effect of soil compaction is the soil moisture correction given by Fecan et al.^45^ parameterisation. However, this has not been possible to explore since current soil reanalysis models (e.g., ERA-Interim Reanalysis, from which our dataset comes) do not account for a real and updated volumetric soil moisture once a volcanic deposit is emplaced. Multiple dust emission schemes have been successfully applied to model the removal of fine ash particles suspended in the atmosphere^17^. However, modelling resuspension of fine particles for such a long time series (2011 to 2016) and a widespread area ($\sim$15 $\hbox {km}^2$) is currently not possible due to computational constraint. Notwithstanding, even when we cannot quantify the compaction of the parental deposit and the loss of fine particles, we propose a simple and reproducible model that allows for the reconstruction of the mass flux temporal decay accounting for all the key processes involved in the depletion of material in a real case. This empirical model is based on the temporal variation of the erodibility $\sigma (t)$, which decreases with time from an initial erodibility $\sigma _0$ (in this case, 0.21) with a rate proportional to the integral of the mass fluxes in the preceding period $[t_0 - t]$ (i.e., $\int _{t_0}^t Q_{slm}\,dt$, where $t_0$ is a reference time, Eqs. 3 and 4). The constant of proportionality $\alpha$ can be then empirically determined (in this case, its optimal value is 1.3 x 10$^{-2}$ m $\hbox {kg}^{-1}$, Fig. 6b). This model provides a very good agreement between the measured *Q*$_{msd}$ and the theoretical *Q*$_{slm}$ streamwise mass fluxes, considering a supply-limited system (Fig. 6a). In addition, the model is able to reproduce the mass flux peaks for specific sampling periods (i.e., August 2011, December 2011, January 2013 and September 2013, Fig. 6a grey curve), which are not recognizable when considering only meteorological fluctuations (Fig. 6a green curve).

This study provides significant insights into the temporal variation of remobilised ash, characterised by a two-phase process that can be empirically modelled. We can expect the most impactful first phase (associated with high mass fluxes of remobilised volcanic ash) to occur within a few months after the eruption, and a long-lasting second phase with exponentially decreasing mass fluxes to occur up to years and decades later. These results have important implications for risk reduction of active volcanic regions. Mitigation measures at short, medium and long term should be considered, particularly for regions that could be potentially affected by significant deposition of fine ash and strong winds, and characterised by large flat areas not protected by topography or vegetation, as it is the case of the Argentinian Patagonia.

## Conclusions

Aeolian erosion of volcanic particles is a stochastic, complex and extremely dynamic process, whose timescales can vary from days to millennia after eruptions. This study provides a comprehensive analysis of supply-limited processes which are very common in natural environments (e.g., loess deposits, sand dunes, volcanic deposits). Understanding these processes and their effect over the long-term on the environment and the communities exposed to volcanic eruptions is extremely important. It is difficult to constrain the interactions between all the involved parameters and accurately determine which are the most relevant for controlling the mass flux decay over time. Our results suggest that compaction processes and the loss of fine material with time, which ultimately limits the supply of particles, play dominant roles on the timescales of aeolian transport of fresh volcanic deposits. Since these processes are difficult to quantify numerically, we propose an exponential model based on the temporal evolution of the erodibility, the factor accounting for the potential of the surface to be eroded, and a proportionality constant (Eqs. 3 and 4). Such a simple and reproducible model for supply-limited systems has never been developed in mineral or volcanic erosion studies. This proposed empirical model is able to describe the depletion of particles due to aeolian activity, under the conditions of the studied volcanic area. Further investigations are needed to test the suitability of this model under different conditions. We have also shown that, in the case of volcanic eruptions where a large volume of material is suddenly injected into the aeolian-transport system, the timescales of mass flux decay, as a function of particle size, follow the characteristic U-shaped curve; such a trend relates the threshold friction velocity to particle size. The proposed methodology represents an innovative approach to link field measurements with saltation theory. However, further studies of supply-limited volcanic systems are necessary to better understand which parameters contribute the most to remobilisation of volcanic ash. Finally, it is important to stress that the field measurements and techniques necessary to characterise ash-remobilisation deposits and processes are not the same as those used to investigate primary pyroclastic deposits. To address this, a detailed uncertainty analysis is presented in the Methods and Materials section. Future work should focus on the development and implementation of field, experimental and modelling strategies to assess and account for all the relevant parameters (e.g., soil moisture, soil compaction and cohesive forces, soil bulk density, precipitation rate thresholds, surface roughness).

## Materials and methods

### Chronology of the 2011–2012 CC eruption

After 41 years of repose, the Cordón Caulle volcano (Chile) erupted on 4th June 2011^20,49,58^. The eruption produced plumes with heights up to 12 km above the vent and generated about 1 $\hbox {km}^3$ of rhyolitic tephra, mostly during the first 15 days of eruption, that was dispersed towards the Argentinian Andes and the Patagonian steppe by prevailing westerly winds. Long-lasting eruptive dynamics combined with strong and shifting winds produced a complex primary tephra-fallout sequence of deposits over a wide area^50–52^. The main primary tephra-fallout deposit consists of 4 main units subdivided into 13 layers^52^. Unit I, that corresponds to the climactic phase (4 and 5 June), was characterised by plumes 9–12 km high above the vent, that dispersed to the SE and generated a lapilli-bearing bed set with a total volume of ca. 0.75 $\hbox {km}^3$^52^. Unit II corresponds to a variable wind direction period when plumes of 6-10 km above the vent, dispersed abruptly to the N and SE, producing a lapilli-bearing deposit of a total volume ca. 0.21 $\hbox {km}^3$. Unit III was primarily produced and sedimented mainly from the 6 to 15 June and was characterised by fluctuating plume heights that dispersed to the E-ESE, and generated a fine-grained deposit consisting of 5 layers (K1 to K5)^50,52^. The volume of the K2 layer was estimated to be ca. 0.05 $\hbox {km}^3$^52^. Unit IV was deposited after the 15th June, and is composed of a millimetres-thick fine ash deposit and is found only in very proximal areas (< 20 km from the vent). On 20 June 2011, an effusive phase started with the emission of viscous lava that lapsed until mid-2012 although its emplacement continued until January 2013^59^. Similar to the case of the 1991 Hudson eruption (Chile), one of the most important long-term consequences of this eruption has been the ash wind-remobilisation^14,20,22^, particularly in the arid and semi-arid regions of the Patagonian steppe. Given the associated stratigraphic position, the fine-grained material and the widespread sedimentation, Unit III represents the Unit most affected by remobilisation in the Argentinian region^23^.

### Sampling strategy and data collection

In this study we used dedicated samplers (BSNE type^21^) installed in 2011 by the National Agricultural Technology Institute of Argentina (INTA) as part of the National Soil Research Program, to systematically collect horizontal airborne material syn-deposition^60^. These collectors, located in 4 strategic erosion sites (Fig. 2), consist of a wind-oriented upright with a weather vane and 3 samplers at fixed heights, 0.15 m, 0.5 m and 1.5 m (Fig. 3a). Based on the same dataset, Panebianco et al.^21^ thoroughly analysed the effects of topography, landscape geomorphology and vegetation cover on the total mass flux; in addition, Dominguez et al.^23^ studied the physical features of particles (i.e., grainsize and shape) to infer information on the transport mechanisms involved. Here, we investigate the timescales of mass flux through a comprehensive analysis of the mass flux decay timescales as a function of particle size and height of sampling collection of the site S4, accounting for a total of 42 samples (14 collection periods at three different heights). Since the studied area is difficult to access, the periods between sample collections are not constant, and therefore the temporal resolution of our dataset varies from about 1–20 months (See details in the ESM-Table S3). Meteorological and soil parameters (i.e., direction and velocity of wind, total precipitation, volumetric soil moisture and roughness length) have been obtained from the ERA-Interim reanalysis data at surface (i.e., 10 m height) of the ECMWF dataset (European Center for Medium-range Weather Forecasts).

#### Grainsize analysis

Granulometry analyses were performed with a laser diffraction particle size analyser (CILAS 1180) at the University of Geneva. It is important to note here that these are disturbed samples due to the transport from the collector to the laboratory. We apply 60 seconds of ultrasound before measurement in order to disaggregate fine particles, but no chemical processes have been used. Grainsize distributions are expressed in half-phi intervals. The equivalence between phi and metric scales, as well as the representative size value for each interval, corresponding to the mid half-phi value, is presented in the ESM-Table S2.

### Streamwise saltation theory

Streamwise saltation (i.e., horizontal) flux is mainly controlled by two competing forces: aerodynamic forces (i.e., scaled with the wind friction velocity, *u*$_*$) and resistive particle forces (i.e., scaled with the threshold friction velocity, *u*$_{*t}$)^38,39,41,42^.

#### Wind friction velocity

The ultimate force driving wind erosion depends on the transfer of momentum from the atmosphere to the surface, which is described by the momentum flux or the shear stress of the wind^38^. This is related to the wind friction velocity by,

5 $$\begin{aligned} u_* = \sqrt{\frac{\tau _s}{\rho _a}} \end{aligned}$$

where $\tau _s$ is the surface shear stress and $\rho _a$ is the air density (taken to be 1.112 kg $\hbox {m}^{-3}$).

Roughness elements on natural surfaces exert a drag on the flow due to the difference in pressure between the windward and the leeward sides of the element. Consequently, the transfer of momentum from the flow to the surface involves a complex process depending on the viscous and pressure forces on the roughness elements^41^. Near the surface, horizontal wind speed is commonly described by a log profile, under neutral stability conditions. It is accepted though, that above some height *z*, the mean wind profile *U(z)* is given by ^61^,

6 $$\begin{aligned} U(z) = \frac{u_*}{\kappa } \Bigg [ln \left( \frac{z}{z_0} \right) \Bigg ] \end{aligned}$$

where $\kappa$ = *0.4* is the von Karman constant, $z_0$ is the roughness length and depends on the geometric features of roughness elements (e.g., height, width, etc.), and *z* is the height above the surface. Following Stull^61^, this can be re-arranged into:

7 $$\begin{aligned} u_* = \sqrt{C_{DN} \; U^2} \end{aligned}$$

where

8 $$\begin{aligned} C_{DN} = \kappa ^2 \left[ \ln \left( \frac{z}{z_0} \right) \right] ^{-2} \end{aligned}$$

We can obtain the forecast roughness length, $z_0$, from the ERA-Interim dataset, taking *z* = 10 m.

#### Threshold friction velocity

Based on the models of Owen^39^ and Iversen et al.^43^, Shao and Lu^44^ derived a simple expression for the threshold friction velocity as a function of particle size, *d*, as follows,

9 $$\begin{aligned} u_{*t} (d) = \sqrt{{f (Re_{*t}}) \; \left( \frac{\rho _p \; g \; d}{\rho _a} \; + \frac{\gamma }{\rho _a \; d} \right) } \end{aligned}$$

where *f (Re* $_{*t}$) is a function of particle Reynolds number at the threshold friction velocity (0.0123 from fitted experiments^44^), $\rho _p$ is the particle density (in kg $\hbox {m}^{-3}$), $\rho _a$ is the air density (1.112 kg $\hbox {m}^{-3}$), *g* is the gravity constant (9.81 m $\hbox {s}^{-2}$) and $\gamma$ is a constant accounting for inter-particle cohesion. Shao and Lu^44^ treat $\gamma$ as a free parameter and fit the Eq. (9) to data obtained from wind-tunnel experiments^43^ and found acceptable agreement for $1.65 \times 10^{-4}< \gamma < 5 \times 10^{-4}$ kg $\hbox {s}^{-2}$. However, there is not yet reliable experimental data to estimate $\gamma$ for *d* < 50 $\upmu$m^41^. According to the Eq. (9), *u* $_{*t}$ is dependent on the particle density, but the density of vesicular particles produced by volcanic eruptions can vary considerably as a function of grainsize^62,63^. Consequently, highly vesiculated large pumices have lower densities compared to small ash particles with lower vesicularity. Density studies suggest that for particles finer than 6$\phi$, the density is approximately constant and fragments can be considered as dense as the skeletal density, without vesicles (i.e., dense rock equivalent, DRE)^62,63^. In order to account for this density variation, here we linearly interpolate the density from 1$\phi$ (i.e., 1270 kg $\hbox {m}^{-3}$ for K2 fragments) to 6$\phi$ (i.e., 2690 kg $\hbox {m}^{-3}$, DRE) using measurements from Pistolesi et al.^52^ (See ESM-Table S4).

The threshold friction velocity is strongly dependent on the soil moisture and vegetation cover. In this study, following Folch et al.^17^, we apply a correction factor accounting for soil moisture, *f* $_w(w)$, according to the parameterisation of Fecan et al.^45^, as follows,

10 $$\begin{aligned} u_{*t} (d, w) = u_{*t} (d) \; f_w(w) \end{aligned}$$

where

11 $$\begin{aligned} f_w(w) = {\left\{ \begin{array}{ll} 1 &{}\quad \text {if } \, w_g \le w' \mathrm{(dry \, soil)}\\ \sqrt{1\; + 1.21 \; (w_g - w')^{0.68}} &{}\quad \text {if} \, w_g > w' \mathrm{(wet \, soil)} \end{array}\right. } \end{aligned}$$

where *w*$_g$ is the gravimetric soil moisture, and *w*$'$ is the maximum amount of water that can be adsorbed. The gravimetric soil moisture is related to the volumetric soil moisture *w* through *w*$_g$ = *w* $\rho _w$/$\rho _b$, with *w* being obtainable from the ERA-Interim dataset; and $\rho _w$ and $\rho _b$ being the water and soil bulk densities (here we use 998 kg $\hbox {m}^{-3}$ and 900 kg $\hbox {m}^{-3}$^51^, respectively). We used a parameterisation for *w*$'$ depending on the clay content *Cs* (i.e., particles $< 2 \upmu$m), according to the expression, *w*$'$ = 0.0014 ${Cs}^2$ + 0.17 *Cs*^45^. Since average clay content of the primary distal deposit is 4.13%^23^, *w*$'$ is 0.7%.

#### Streamwise mass flux of particles

The streamwise (horizontal) mass flux *Q* is defined as the vertical integral of the saltation streamwise mass flux density *q* at each given height *z*^38–42^ and can be expressed as,

12 $$\begin{aligned} Q = \int q(z) dz \end{aligned}$$

For field measurements, the average mass flux has been calculated according to,

13 $$\begin{aligned} q_z = \frac{m_z}{A \; dt} \end{aligned}$$

where $$q_z$$ is the time-averaged mass flux at a given height *z* (units of kg $$\hbox {m}^{-2}$$$$\hbox {day}^{-1}$$), $$m_z$$ is the mass measurement at each height; A = 1 × 10$$^{-3}$$$$\hbox {m}^2$$ is the surface area of the collector opening and *dt* is the number of days per period of collection. We fit a linear relationship of the form *mz + n*, the measured streamwise *Q*$$_{msd}$$ is then given by

14 $$\begin{aligned} Q_{msd} = \int _0^{1.5} (m\ z + n) \,dz = \left[ \frac{m\ z^2}{2} \, + \, n\,z \right] \Big |_0^{1.5} \ \end{aligned}$$

The theoretical streamwise mass flux, *Q*$_{th}$, has been calculated according to the parameterisation of Shao et al.^40^ based on the model of Bagnold-Owen^39^ (Eq. 15). Additionally, we also imposed two meteorological conditions; first, the total precipitation rate *p* must not exceed a threshold $p_t$ = 0.01 mm $\hbox {h}^{-1}$, following Leadbetter et al.^30^, and second, the wind friction velocity $u_*$ must exceed the threshold friction velocity $u_{*t}$ , such that

15 $$\begin{aligned} Q_{th} (d) = {\left\{ \begin{array}{ll} 0 &{} \text {if} \, p \ge p_t \; \mathrm{or} \; u_*< u_{*t} (d) \\ C_o \, \frac{\rho _a \, u_*^3}{g} \, \Bigg ( 1 - \frac{u_{*t}^2 \ (d)}{u_*^2} \Bigg ) &{} \text {if} \, p \,< p_t \; \mathrm{and} \; u_* \ge u_{*t} (d) \end{array}\right. } \end{aligned}$$

where *C*$$_o$$ is the dimensionless Owen Coefficient, typically close to 1^40^. *Q*$$_{th}$$ is expressed in kg $$\hbox {m}^{-1}$$$$\hbox {s}^{-1}$$.

This theoretical framework allows us to calculate *Q*$_{th}$ for uniformly sized particles; however, volcanic primary deposits have dispersed size distributions. Since the effects of both particle interactions and sorting of natural soils are very complex, we follow Shao et al.^46^ and assume that *Q*$_{th}$ is not significantly altered by the presence of a range of particle sizes. Consequently, we calculate *Q*$_{th}$ for each particle size fraction $d_i$, from the mass fraction, $f_i$, of that particular size class in the parent soil (i.e., the distal tephra-fallout grainsize distribution). The total streamwise mass flux is given then by the sum of all size fractions,

16 $$\begin{aligned} Q_{th} = \sum Q_{th}(d_i) \; f_i \end{aligned}$$

### Fitting procedure for the double exponential decay

Mass flux decay has been analysed under the hypothesis of a double exponential dependence in time (Eq. 2). The uncertainty associated with the four fitted parameters (i.e., $\tau _1$, $\tau _2$, *b, c*) is determined by the analysis of the non-linear least squares method, imposing a confidence interval of 68$\%$ (i.e., 1 standard deviation) in the Matlab fitting toolbox. A comprehensive analysis for each size class has been done including the independent evaluation of each of the two terms of the double exponential combined with the error bars derived from this procedure. We highlight two particular cases: (i) particles larger than 180 $\upmu$m are practically not present in the primary deposit (see ESM-Table S4) but they are found in the samplers, either because they are coarser volcanic particles coming from the prevailing westerly winds with time (CC primary proximal deposit is coarser than distal one^23^), or because they are old soil sediments. As a consequence, the mass flux decay of these size-classes is not consistent within this study and they are not shown in the Fig.  4. (ii) particle sizes of 26 to 53 $\upmu$m show strongly pronounced peak and a rapid decay that $\tau _1$ is not able to describe. As a result, error bars associated with these classes are considerably larger than other classes, and, in the case of 1.50 m the fit does not converge for $\tau _1$. It is important to notice that these classes are the most abundant in volume in the primary deposit (ESM-Table S4), hence it is not surprising that the first mass flux peaks were quite strong.

### Uncertainty analysis

Aeolian erosion of volcanic particles is a very dynamic process. Although meteorological seasonality has been considered (based on the ECMWF ERA-Interim Re-Analysis dataset), several uncertainties, particularly associated with the volumetric soil moisture estimation, *w*, arose from the reanalysis model. On the other hand, due to lack of data, we have taken soil parameters to be static (i.e., initial values of primary deposit: soil bulk density, $\rho _b$, and average clay content, $C_s$), neglecting changes due to seasonal variations, stochastic wind fluctuations and compaction effects. One of the major caveats in this study is the irregular and large time window of sample collection. The Patagonia steppe is a remote area of difficult access and it is not feasible to establish a more systematic sampling. For future studies, we strongly recommend both a temporal resolution of the sampling suitable for the possible mass flux decay and a regular monitoring of physical conditions of the parent soil surrounding the dust-collectors (e.g., soil density, moisture, thickness, grainsize). Table 1 summarises the main caveats associated with the analysed parameters. Given the complexity of aeolian dynamics due to the turbulence of gusty winds and the effect of roughness elements (e.g., vegetation), these factors have not been considered in this study.

**Table 1:** Caveats concerning the estimation of the parameters required for the calculation of the threshold friction velocity *u*$_{*t}$ and the streamwise mass flux *Q*$_{th}$.

Parameter	Abbreviation	Units	Caveats
Cohesive forces^44^	$\gamma$	kg $\hbox {s}^{-2}$	This term considers cohesive forces linearly proportional to particle diameter, however, van der Waals, electrostatic, liquid and chemical forces, which are sensitive to soil properties (e.g., particle shape, surface texture, mineralogy, deposit sorting, bonding agents, such as moisture and soluble salts) are not considered^44^. All these parameters are very important in order to account for compaction of primary volcanic deposits.
Soil bulk density	$\rho _b$	kg $\hbox {m}^{-3}$	This is probably the most important parameter changing over time. It is strongly related to the infiltration of water and thus soil moisture. It could indirectly account for compaction of the deposit. In this study, it is considered constant and corresponds to the primary deposit (Unit III) density at the beginning of the eruption.
Volumetric soil moisture	*w*	$\%$	It is obtained from soil models that are not updated once the eruption occurs. From the ECMWF dataset, “volumetric soil moisture layer 1” corresponds to 1-7 cm depth. However, it is only the most superficial layer (few mm) and generally much drier than the one to be remobilised.
Max. amount of water that can be adsorbed	*w’*	$\%$	Only based on the clay content^45^, it does not account for infiltration or permeability of volcanic particles.
Precipitation rate threshold	*p*$_t$	mm $\hbox {h}^{-1}$	The precipitation threshold inhibiting remobilisation processes is not well constrained. Here we use 0.01 mm $\hbox {h}^{-1}$, following the UK Met Office modelling strategy^30^.
Particle density	$\rho _p$	kg $\hbox {m}^{-3}$	Particle density is strongly dependent on the grainsize and vesicularity, which is particularly important in volcanic particles. It is difficult to measure density for particles smaller than $1\phi$. There are linear^62^ and sigmoidal models^63^ to fit density distributions. Here we use a linear model from 1$\phi$ particles and DRE measurements.
Roughness length	*z*$_0$	m	The abrupt change in surface roughness is not considered by the atmospheric forecast models. However, it is strongly dependent on the large amount of loose particles on the surface due to fresh volcanic deposits. It has important implications on the wind friction velocity. Here we use the forecast surface roughness of the ERA-Interim Re-Analysis dataset.

## Supplementary information

Supplementary Information.
