# Supplementary material for: Mass flux decay timescales of volcanic particles due to aeolian processes in the Argentinian Patagonia steppe
Source: Sci Rep. 2020 Sep 2;10:14456. doi: 10.1038/s41598-020-71022-w (PMC7468302; doi:10.1038/s41598-020-71022-w)
Supplement: Supplementary file 1 — Supplementary Information. [file 41598_2020_71022_MOESM1_ESM.pdf]

Supplementary information for: Mass flux decay timescales of volcanic particles due to aeolian processes in the Argentinian Patagonia steppe

Lucia Dominguez[1,*], **Eduardo Rossi**[1], **Leonardo Mingari**[2], **Costanza Bonadonna**[1], **Pablo Forte**[3], **Juan Esteban Panebianco**[4], **and Donaldo Bran**[5]

[1]Department of Earth Sciences, University of Geneva, Geneva, Switzerland
[2]Barcelona Supercomputing Center, Barcelona, Spain
[3]Instituto de Estudios Andinos (IDEAN) (UBA – CONICET), Buenos Aires, Argentina
[4]INCITAP (Institute for Earth and Environmental Sciences, CONICET) and Facultad de Ciencias Exactas y Naturales, UNLPam, Santa Rosa, Argentina
[5]Institute of National Agricultural Technology-INTA, Bariloche, Argentina
[*]Lucia.Dominguez@unige.ch

1 Wind-remobilisation of the tephra-fallout deposit associated with 2011-2013 Cordón Caulle eruption: spatial distribution

Here we analyse the spatial distribution of ash remobilisation based on data collection of airborne material at sites S2, S3, S4 and S6 (Fig. S1a). Generally, the mass flux of remobilised material is expected to be inversely proportional to the collector height[1,2]. Despite some exceptional cases that are treated in depth by Panebianco *et al.*[3] based on the same dataset, the highest mass fluxes are indeed found closest to the ground (i.e. 0.15 m) as it is expected (Fig. S1b-e). The smallest average mass flux (¡ 0.75 kg m^{-2} day^{-1}) was recorded in the western-most site (S2), where the primary deposit is composed mainly of coarse ash (76% in volume of particles between 63 μm and 2 mm[4]), with ∼1 cm thick (Fig. S1a). Substantially, larger values of mass flux (up to 2.25 kg m^{-2} day^{-1}) are recorded in the south collector (S6), where the primary deposit is similar in thickness (∼1 cm, Fig. S1a) but composed of fine-ash (83% vol. of particles smaller than 63 μm[4]). Although tephra-fallout deposits generally decrease in thickness and grainsize with distance from the vent, complex sedimentation processes can generate secondary thickness maxima, which is the case of Unit III of the 2011-CC deposit (Fig. S1a)[4]. Consequently, the largest average mass fluxes were recorded at sites S3 and S4, where the primary tephra-fallout deposit is thicker (2-3 cm, Fig. S1a).

Figs. S1b-e show that the average mass flux peak does not occur simultaneously for all the sites. Indeed, the first (and largest) mass flux is recorded at S4 on 2 August 2011, two months after the beginning of the eruption (Fig. S1c). Unfortunately, there is no data for the site S3 prior to the collector installation on 3rd August 2011. Consequently, the first peak in our dataset for this site occurs on 6th December 2011 (Fig. S1d). Interestingly, the first peak at the site, S6, only occurs on 23rd September 2011 (Fig. S1e). In contrast, the first peak at the western-most site, S2, was recorded on 11th November 2011, five months after the eruption (Fig. S1b). Figs. S1b-e also show that the mass flux decays non-monotonically over time. In fact, there are at least two further mass flux peaks recorded for all of the collectors on 6th December 2011 and on 29th January 2013; a third peak is also recorded at S2 and S3 on 16th January 2014. Additionally, a peak is recorded at S4 on the 18th September 2013. All the collectors show a monotonic decay in mass flux after April 2014 (Fig. S1b-e).

The observed behaviour depends on the regional variation of primary deposit features, such as thickness, volume, grainsize and particle densities, as well as local aspects (e.g. topography, vegetation, exposure to wind). We observed the spatial variation of mass fluxes along a NW-SE transect (Fig. S1a) and found that the lowest mass fluxes occur in the proximal area (close to San Carlos de Bariloche, site S2) and are associated with limited availability of erodible fine particles (thin deposit, coarse grainsize[4]). In contrast, the highest mass fluxes are found in medial areas (NE of Ingeniero Jacobacci, sites S3 and S4) and this could be related to the large availability of fine particles, due to the secondary maximum in thickness of the primary Unit III (thick deposit, fine grainsize[4]). Additionally, as stated by Panebianco *et al.*[3], these high values of mass flux are also associated with little vegetation cover, convex landscapes (e.g. S4), and a flat topography (e.g. S3), where a wide and open valley extends towards the SE and important erosion processes occur[3],[5]. Finally, low mass fluxes were recorded in the western site (S6) where fine particles are present but not in the same quantities than in medial areas (thin deposit, fine grainsize[4]). These results demonstrate that the intrinsic characteristics of the primary pyroclastic deposits, such as volume (and associated thickness spatial variations) and granulometry are key factors controlling the spatial distribution of ash-remobilisation once an eruption occurs.

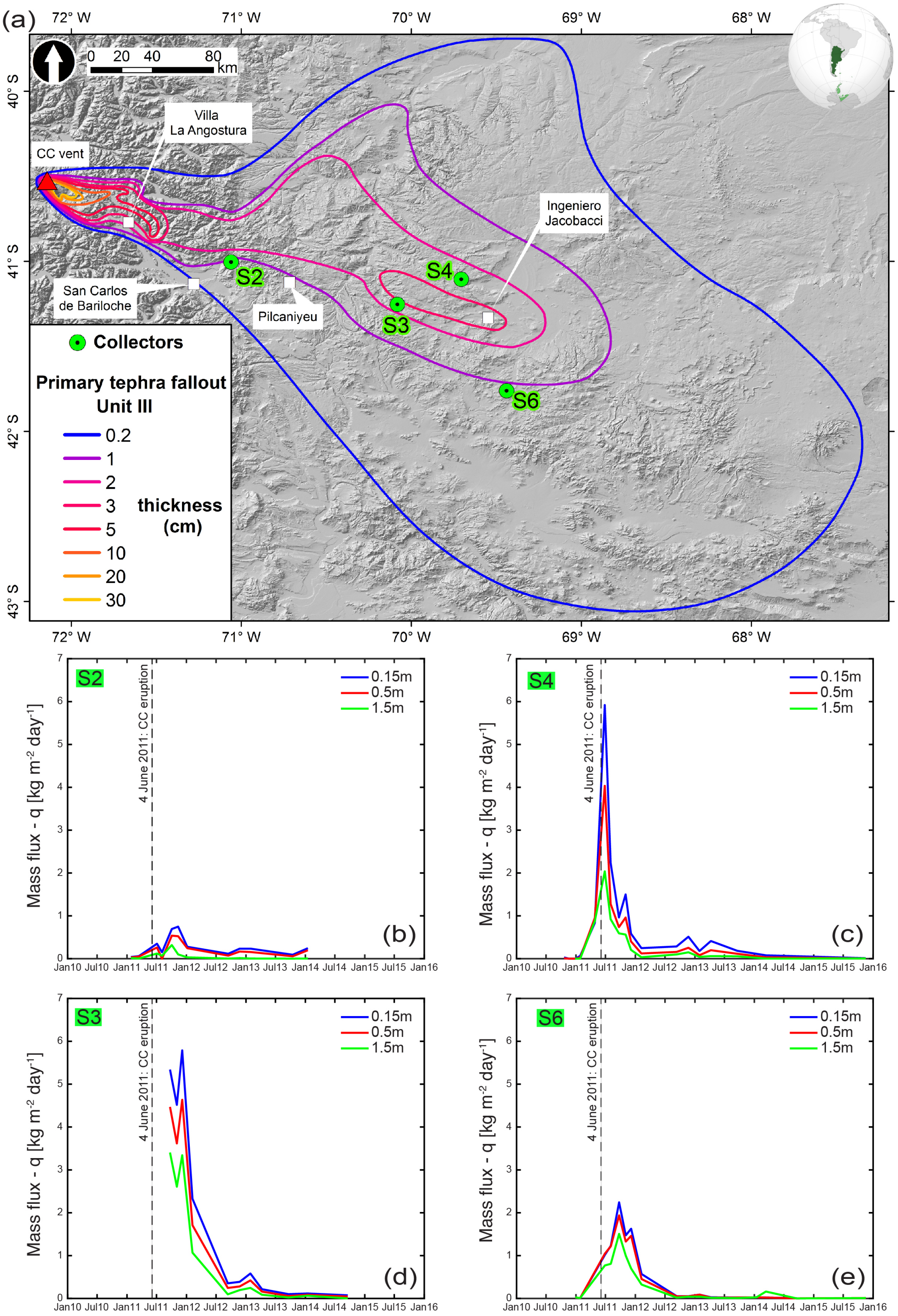

Figure S1. **(a)** Isopach map of the primary Unit III in cm (data from Dominguez *et al.*[4]). Sampling collectors are also shown. **(b-e)** Mass flux measured at 4 selected sites, S2, S3, S4 and S6 for 3 fixed heights (blue: 0.15 m, red: 0.50 m, green: 1.50m). For comparison, pre-eruption mass fluxes are also shown at collectors S2, S4 and S6 (S3 was only installed on 3rd August 2011, 2 months after the beginning of the eruption). Dashed lines correspond to the CC-eruption starting date. Map created by the authors using ArcGIS software by Esri (version 10.3 copyright 1995-2015) used herein under license. Hillshade generated from the Global Multi-resolution Terrain Elevation Data (GMTED2010) available in https://earthexplorer.usgs.gov. Globe image available in https://www.pngegg.com/en/png-yzcom.

Temporal variation in ash-remobilisation processes is the result of a complex interaction between surface features and meteorological conditions over time. The studied area is characterized by a distinct climate gradient with contrasting annual precipitations ranging from ∽500 mm year^{-1} in the western sub-humid steppe (S2 site), to 150-200 mm year^{-1} in the eastern arid steppe (S3, S4, S6 sites)[5]. Consequently, remobilisation is generally restricted in western areas and enhanced in the east[4]. Analysis of mass flux peak values (Fig. S1b-e) shows that major remobilisation occurred some time after the eruption once primary sedimentation processes had completed. Additionally, the eruption started in the middle of winter when high values of precipitations, relative humidity and soil moisture, inhibit aeolian erosion. It is notable that, even with similar weather conditions at both sites, the first peak in mass flux at S4 was recorded in early August 2011 (Fig. S1c), two months after the beginning of the CC eruption, during the Austral winter, whilst at S6 the first peak was only recorded in September 2011 (Fig. S1e). However, the availability of fine material is larger at S4 due to the secondary maximum in thickness. In contrast, the first peak in the western sub-humid steppe (S2) was not recorded until mid-November 2011 (Fig. S1b), five months after the eruption, once strong winds and low precipitations occurred during spring. An important turning point towards a monotonic depletion of mass fluxes since 2014 is associated with a strong precipitation event occurred on 2-8 April 2014.

2 Summary of parameters used in this study

Abbreviation	Parameter	Units
a, b, c	exponential fitting parameters	kg m^{-2} day^{-1}
A	surface area of the collector opening	m^2
C_o	Owen coefficient	dimensionless
C_s	clay content	%
d	particle size	m
d_i	particle size fraction i	m
f_i	mass fraction of the particle size fraction i	dimensionless
g	gravity constant	m s^{-2}
m_z	mass measured at height collector z	kg
m	linear fitting parameter	kg m^{-3} s^{-1}
n	linear fitting parameter	kg m^{-2} s^{-1}
p	precipitation rate	mm h^{-1}
p_t	threshold precipitation rate	mm h^{-1}
q	streamwise mass flux density (in this paper, mass flux)	kg m^{-2} s^{-1}
Q	vertically-integrated streamwise mass flux (in this paper, streamwise mass flux)	kg m^{-1} s^{-1}
Q_{th}	theoretical streamwise mass flux	kg m^{-1} s^{-1}
Q_{msd}	measured streamwise mass flux	kg m^{-1} s^{-1}
Q_{slm}	supply-limited streamwise mass flux	kg m^{-1} s^{-1}
$f(Re_{*t})$	function of particle Reynolds number	dimensionless
t	time	s // days
t_0	integrative reference time	s
U	wind velocity	m s^{-1}
u_*	wind friction velocity	m s^{-1}
u_{*t}	threshold friction velocity	m s^{-1}
w_g	gravimetric soil moisture	%
w	volumetric soil moisture	%
w'	maximum amount of water that can be adsorbed	%
z	height	m
z_0	roughness length	m
α	constant - erodibility model	m kg^{-1}
γ	constant associated with inter-particle cohesion	kg s^{-2}
κ	von Karman constant	dimensionless
ρ_a	air density	kg m^{-3}
ρ_b	soil bulk density	kg m^{-3}
ρ_p	particle density	kg m^{-3}
ρ_w	water density	kg m^{-3}
σ	time-varying erodibility	%
σ_0	initial erodibility	%
τ_s	surface shear stress	kg m^{-1} s^{-1}
τ	decay timescale	days
τ_1	decay timescale - phase I	days
τ_2	decay timescale - phase II	days

Table S1. Summary of parameters used in this study.

3 Grain size classes analysed in this study

Size class	Grain size (ϕ)	Grain size range (μm)	Representative size (μm)
1	10.0	1.0 - 1.4	1.2
2	9.5	1.4 - 2.0	1.6
3	9.0	2.0 - 2.8	2
4	8.5	2.8 - 3.9	3
5	8.0	3.9 - 5.5	5
6	7.5	5.5 - 7.8	7
7	7.0	7.8 - 11.0	9
8	6.5	11.0 - 15.6	13
9	6.0	15.6 - 22.0	19
10	5.5	22 - 32	26
11	5.0	32 - 45	37
12	4.5	45 - 63	53
13	4.0	63 - 90	74
14	3.5	90 - 125	105
15	3.0	125 - 180	149
16	2.5	180 - 250	210
17	2.0	250 - 355	297
18	1.5	355 - 500	420
19	1.0	500 - 710	595

Table S2. Grainsize classes analysed in this study expressed in ϕ scale and the corresponding size range in microns. Representative size corresponds to the half ϕ interval, i.e., $1/4\phi$. The size referred in the text and figures corresponds to the "representative" size per each class.

4 Model input parameters

Period of collection	Date start	Date end	Total number of days
1	27-Apr-11	27-Jun-11	61
2	28-Jun-11	2-Aug-11	35
3	3-Aug-11	22-Sep-11	50
4	23-Sep-11	2-Nov-11	40
5	3-Nov-11	5-Dec-11	32
6	6-Dec-11	7-Feb-12	63
7	8-Feb-12	10-Sep-12	215
8	11-Sep-12	22-Nov-12	72
9	23-Nov-12	29-Jan-13	67
10	30-Jan-13	9-Apr-13	69
11	10-Apr-13	18-Sep-13	161
12	19-Sep-13	14-Mar-14	176
13	15-Mar-14	16-Nov-15	611
14	17-Nov-15	03-Oct-16	321

Table S3. Sample collection periods at the site S4.

Grain size (ϕ)	Representative size (μm)	Particle density (kg m^{-3})	Primary GSD (%vol)	Primary GSD (%mass)
10.0	1.2	2690	0.980	0.003
9.5	1.6	2690	1.645	0.513
9.0	2	2690	2.197	3.736
8.5	3	2690	2.697	9.528
8.0	5	2690	3.368	13.496
7.5	7	2690	4.329	13.980
7.0	9	2690*	5.717	13.184
6.5	13	2572	7.873	11.084
6.0	19	2453	10.489	8.721
5.5	26	2335	13.109	6.624
5.0	37	2217	14.642	5.015
4.5	53	2098	14.933	3.902
4.0	74	1980	11.172	3.125
3.5	105	1862	4.660	2.546
3.0	149	1743	0.684	1.906
2.5	210	1625	0.004	1.135
2.0	297	1507	0.000	0.000
1.5	420	1388	0.000	0.000
1.0	595	1270*	0.000	0.000

Table S4. Model input parameters for primary particles. Linear density calculated from 1ϕ to 7ϕ using density values measured by Pistolesi *et al.*[6], and assuming that particles smaller than 7ϕ have a dense rock equivalent (DRE) density, according to Bonadonna and Phillips model[7].

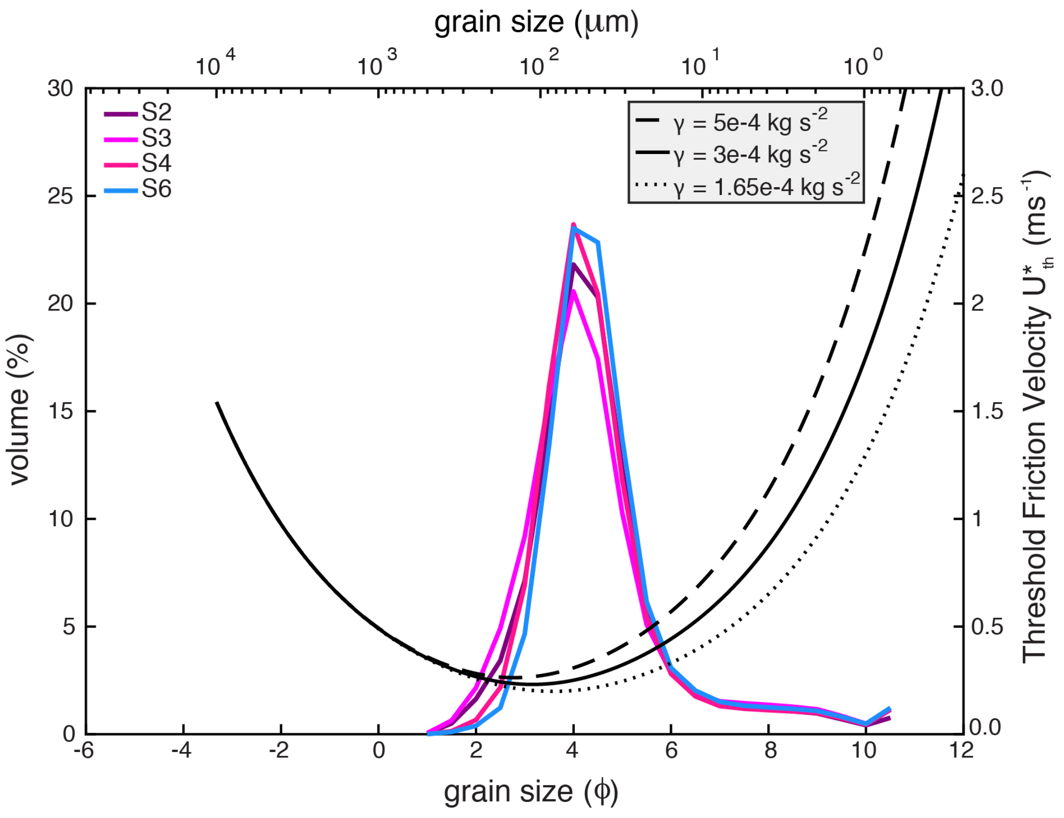

Figure S2. Comparison of grainsize distributions for airborne material and the threshold friction velocity using the Shao and Lu model and different values of γ[8].

References

1. Shao, Y. *Physics and Modelling of Wind Erosion* (Springer, 2008), second revised edn.

2. Arnalds, O., Thorarinsdottir, E. F., Thorsson, J., Waldhauserova, P. D. & Agustsdottir, A. M. An extreme wind erosion event of the fresh Eyjafjallajökull 2010 volcanic ash. *Sci. Reports* **3**, 1257, DOI: 10.1038/srep01257 (2013).

3. Panebianco, J. E., Mendez, M. J., Buschiazzo, D. E., Bran, D. & Gaitán, J. J. Dynamics of volcanic ash remobilisation by wind through the Patagonian steppe after the eruption of Cordón Caulle, 2011. *Sci. Reports* **7**, 1–11, DOI: 10.1038/srep45529 (2017).

4. Dominguez, L. *et al.* Aeolian Remobilisation of the 2011-Cordón Caulle Tephra-Fallout Deposit : Example of an Important Process in the Life Cycle of Volcanic Ash. *Front. Earth Sci.* **7**, 1–20, DOI: 10.3389/feart.2019.00343 (2020).

5. Gaitán, J. J., Ayesa, J. a., Umaña, F., Raffo, F. & Bran, D. B. Cartografía del área afectada por cenizas volcánicas en las provincias de Río Negro y Neuquén. Tech. Rep., National Institute of Agriculture of Argentina (INTA) (2011).

6. Pistolesi, M. *et al.* Complex dynamics of small-moderate volcanic events: the example of the 2011 rhyolitic Cordón Caulle eruption, Chile. *Bull. Volcanol.* **77**, 3, DOI: 10.1007/s00445-014-0898-3 (2015).

7. Bonadonna, C. & Phillips, J. C. Sedimentation from strong volcanic plumes. *J. Geophys. Res. Solid Earth* **108**, 2340 (2003).

8. Shao, Y. & Lu, H. A simple expression for wind erosion threshold friction velocity. *J. Geophys. Res.* **105**, 22.437–22.443 (2000).
